# A Systematic Review on Deep Learning with CNNs Applied to Surface Defect Detection

**DOI:** 10.3390/jimaging9100193

**Published:** 2023-09-25

**Authors:** Esteban Cumbajin, Nuno Rodrigues, Paulo Costa, Rolando Miragaia, Luís Frazão, Nuno Costa, Antonio Fernández-Caballero, Jorge Carneiro, Leire H. Buruberri, António Pereira

**Affiliations:** 1 Computer Science and Communications Research Centre, School of Technology and Management, Polytechnic of Leiria, 2411-901 Leiria, Portugal; esteban.c.cumbajin@ipleiria.pt (E.C.); nunorod@ipleiria.pt (N.R.); paulo.costa@ipleiria.pt (P.C.); rolando.miragaia@ipleiria.pt (R.M.); luis.frazao@ipleiria.pt (L.F.); nuno.costa@ipleiria.pt (N.C.); 2 Instituto de Investigación en Informática de Albacete, 02071 Albacete, Spain; 3 Departamento de Sistemas Informáticos, Universidad de Castilla-La Mancha, 02071 Albacete, Spain; 4 Grestel-Produtos Cerâmicos S.A, Zona Industrial de Vagos-Lote 78, 3840-385 Vagos, Portugal; jorgecarneiro@grestel.pt (J.C.); leireburuberri@grestel.pt (L.H.B.); 5 INOV INESC Inovação, Institute of New Technologies, Leiria Office, 2411-901 Leiria, Portugal

**Keywords:** defect detection, deep learning, CNN, industrial surface, automatic surface inspection, quality inspection

## Abstract

Surface defect detection with machine learning has become an important tool in industries and a large field of study for researchers or workers in recent years. It is necessary to have a simplified source of information that helps us to better focus on one type of surface. In this systematic review, we present a classification for surface defect detection based on convolutional neural networks (CNNs) focused on surface types. Findings: Out of 253 records identified, 59 primary studies were eligible. Following the Preferred Reporting Items for Systematic Reviews and Meta-Analyses (PRISMA) guidelines, we analyzed the structures of each study and the concepts related to defects and their types on surfaces. The presented review is mainly focused on finding a classification for the types of surfaces most used in industry (metal, building, ceramic, wood, and special). We delve into the specifics of each surface category, offering illustrative examples of their applications within both industrial and laboratory settings. Furthermore, we propose a new taxonomy of machine learning based on the obtained results and collected information. We summarized the studies and extracted the main characteristics such as type of surface, problem types, timeline, type of network, techniques, and datasets. Among the most relevant results of our analysis, we found that the metallic surface is the most used, as it is the one found in 62.71% of the studies, and the most prevalent problem type is classification, accounting for 49.15% of the total. Furthermore, we observe that transfer learning was employed in 83.05% of the studies, while data augmentation was utilized in 59.32%. Our findings also provide insights into the cameras most frequently employed, along with the strategies adopted to address illumination challenges present in certain articles and the approach to creating datasets for real-world applications. The main results presented in this review allow for a quick and efficient search of information for researchers and professionals interested in improving the results of their defect detection projects. Finally, we analyzed the trends that could open new fields of study for future research in the area of surface defect detection.

## 1. Introduction

Defect detection is an important part of industrial processes. Currently, many manual inspections are carried out with experts in the process but have a high cost due to the staff’s working hours. In recent years, there has been a significant increase in the use of machine learning to carry out these processes, reaching a significant impact on industries to improve the quality of their products. Within the inspection process in an industry, the detection of defects has a very important role because it approves or rejects parts produced in factories or delivered by suppliers. It also helps to reduce material wastage because it can include the rework and repair of parts [1], even though within machine learning there are several options for solving defect detection problems, such as support vector machines (SVMs) in the metal industry [2], cellular neural networks (CNNs) in the metal industry [3], or using different image processing algorithms in the metal industry [4]. Based on the information collected in [5], CNNs stand out in a number of existing studies, result in the extraction of information from images, and outperform other traditional machine learning architectures; therefore, CNNs were chosen as our starting point. CNNs have mostly been used for defect detection in metals and recently in other materials or surfaces such as wood, ceramics, and concrete, among others. Currently, there are types of defects that cannot be detected by various factors, so there is no final or specific solution for the target detection task. The most popular algorithms are grounded on deep learning methods because they are based on input data, so they automatically learn the characteristics of the defects. Conversely, traditional detection technology is based on human labor, so the difference is that current methods reduce labor consumption [6]. Defect detection is generally carried out on images of a dataset from a camera, but it can also be developed using lamb wave data converted images such as in [7] or through sensors such as in [8], where the authors obtain C-scan images from an anisotropic magneto-resistive (AMR) sensor. There are more examples to generate a dataset, but this review is focused on learning through images because visual inspections are carried out in industrial processes and the objectives of the studies found is to improve said processes. In addition, large amounts of information and datasets were found, which helped us to better understand how the creation and use of images in CNNs have evolved. According to He et al. [9], using deep learning is possible for learning directly from two-dimensional images and for reducing image preprocessing; for these reasons, there is no need to manually extract features, since they are automatically learned more accurately from input layers. The incorporation of various techniques such as transfer learning (TL) and data augmentation (DA), with utilization rates of 83.05% and 59.32%, respectively, has notably enhanced experimental outcomes. A majority of studies (67.80%) engage in trials using customized CNNs, successfully identifying combinations that elevate the accuracy of current CNN models. Similarly, we detail the various types of datasets and their respective creation methods for each surface, accompanied by real-world examples.

In this article, a CNN literature review is carried out, considering various aspects such as types of surfaces, different types of CNNs, datasets, cameras, and network architectures. It was possible to go from 253 studies to 59 specific studies and perform a systematic review. In this review, defect detection articles (DDAs) use labeled data, so all articles are of the supervised learning type, because the majority of the conducted studies using CNNs use supervised learning; therefore, we exclude other types such as unsupervised learning, self-supervised, semisupervised, and reinforcement.

### 1.1. Research Relevance

The study of surface defect detection has significant relevance in industries because it improves the quality of products and reduces production costs, but as of the date in which this systematic review is carried out, there are still no studies comparing types of surfaces, problem types, or origins of the datasets. This systematic review becomes a significant help for researchers and students who need to focus directly on a type of surface, speeding up the search time and providing a general guide.

### 1.2. Research Questions

To conduct this review, we first conducted a preliminary study to define a group of which we consider to be the most relevant research questions (RQs), which we will answer throughout this article.

- RQ1: Which are the most used types of surfaces in defect detection?
- RQ2: What are the main problem types for surface defect detection?
- RQ3: Which is the type of network architecture most used for each type of surface defect detection?
- RQ4: What techniques were used to improve performance in studies regarding surface defect detection?
- RQ5: Which is the most used type of dataset?
- RQ6: How did the number of studies evolve over the years?

### 1.3. Contributions

Besides the intrinsic value of the answers to the main research questions of this article, several other contributions are highlighted:

- A comparison between techniques is made by the type of material, which will guide researchers when searching specifically for a specific material or to perceive the main trends in the industry;
- The studies were classified by type of learning, to easily understand what is being used in each study reviewed;
- The proposal of a taxonomy for machine learning and surface defect detection.

### 1.4. Review Structure

In Section 2, the methods used in this systematic review are addressed. Then, in Section 3, after the systematic literature review, the obtained results will be presented, showing results through relevant tables and graphs, as well as the taxonomy and existing applications according to the surface type. Section 4 discusses and analyzes the research questions and also highlights the main learned lessons. Finally, Section 5 presents the relevant conclusions from this systematic review and future directions within this field of study.

## 2. Methods

**Figure 1:**
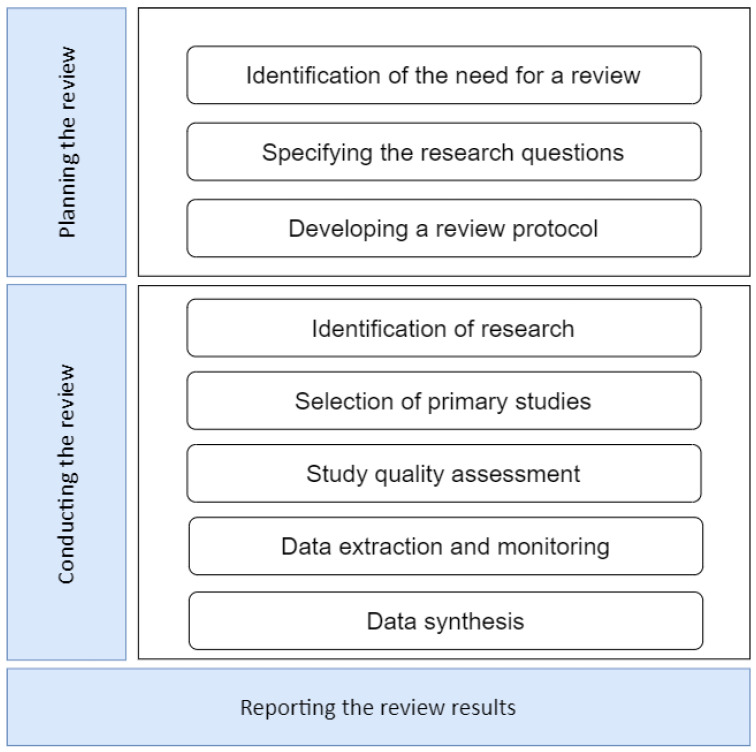
Systematic review process.

This systematic review was based on three stages represented in Figure 1: planning the review, conducting the review, and reporting the review results. For conducting the review in Section 2.3, a thorough literature search was undertaken, employing the PRISMA methodology to meticulously assess and choose pertinent primary studies.

First, planning the review; the need to develop this systematic review was determined, and then we defined the research questions and created a review protocol. Second, conducting the review; with the review protocol implemented, the next step was to conduct the review stage. We started to identify the research questions because these serve as a guide to carrying out this review, and these questions were answered while this systematic literature review (SLR) was developed. Then, we defined search strategies where we found the first research studies to be reviewed, followed by a primary selection of studies that are relevant to our research questions. After that, we proceeded with the study quality assessment, obtaining better filtering. Once the studies were chosen, the next step was data extraction, where the information obtained from the primary studies was recorded; in this case, the work was made easier by using forms to answer the research questions posed at the beginning. Then, the results of the primary studies were collected and summarized (data synthesis). Finally, the results were reported; reporting the review results.

### 2.1. Literature Search

The search was carried out using synonyms and alternative terms referring to the same topic, with a combination of the boolean expressions “AND” and “OR”. In addition, these searches were filtered to find studies between the years 2011 and 2021. We decided to start our search in 2011 until nowadays due to the big boom in deep learning using CNNs that started in 2012 when AlexNet won the 2012 ImageNet Challenge for image classification. AlexNet proved to be a landmark deep learning model with GPU acceleration, triggering the deep learning revolution. Although this network was not the first to use the GPU, the big stage where it succeeded gave it media attention, setting a milestone and sparking the deep learning revolution. These facts gave us a guideline, so we decided to keep a one-year margin (2011) to include possible studies before the rise of AlexNet.

A general key was created as a basis and 559 primary research studies were found in Scopus. Then, the search parameters were improved, and after several attempts, Key1 was created and used to search for studies in electronic digital databases. After performing an analysis of backward and forward citation search, we found relevant articles that guided us to structure the search for studies; surveys especially helped us because they cited articles with relevant topics to our search topic. With this information, Key1 was improved again to focus mainly on surfaces, quality control, defect detection, and machine learning, in a combination that allowed us to find the articles studied in the following stages. Key1 can be used in several electronic journals because the journals share a general format, which facilitates the search process. The four electronic databases used to search for primary studies were Scopus, IEEE Xplore, ACM Digital Library, and Web of Science. Key1 is expressed as follows: ((surface AND (ceramic OR metal OR wall OR wood OR building)) AND (defect OR deformity OR fracture OR deficiency OR crack) AND ((quality control) OR (detection OR detecting OR identification OR sensing OR classification)) AND (cnn OR convolutional OR machine learning OR deep learning)).

The search process begins with the 4 electronic databases and Key1, so searches are performed to find candidate studies, which are then filtered according to the exclusion and inclusion criteria shown in the next section.

### 2.2. Eligibility Criteria

The criteria results considered to assess the results in this study include both inclusion and exclusion criteria, which are presented below.

Inclusion criteria:

- Empirical studies using CNNs for surface defect detection;
- Empirical studies using supervised learning for surface defect detection;
- Empirical studies combining CNNs and other commonly used machine learning techniques;
- Review studies, conference papers, and articles;
- Studies between 2011 and 2021;
- Studies in English;
- Final published versions.

Exclusion criteria:

- Studies without empirical analysis or results of the use of CNNs;
- Studies using CNN techniques in a context other than surface defect detection;
- Studies using CNNs with datasets not based on images;
- Studies with only abstracts;
- Articles in press.

### 2.3. Study Selection

**Figure 2:**
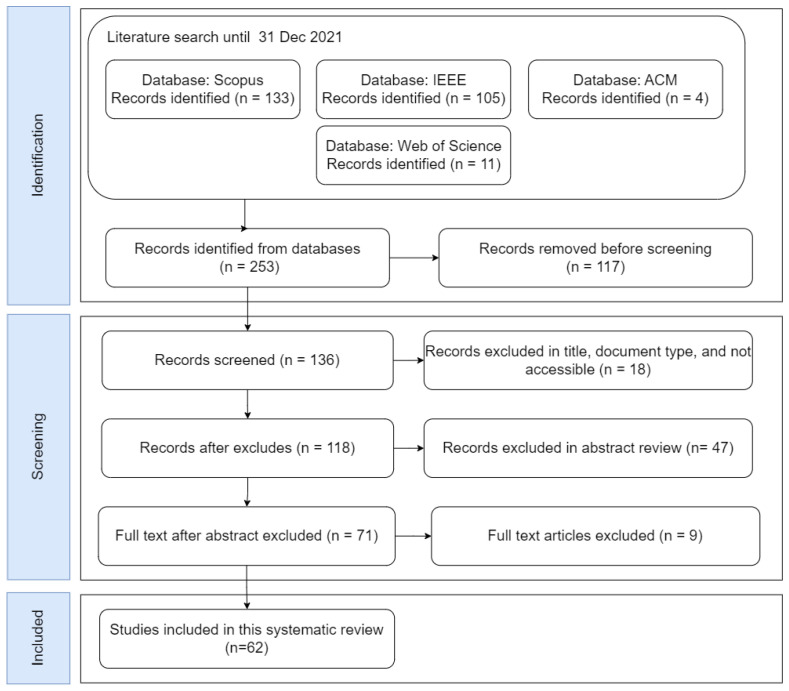
Study selection process.

This study selection is based on PRISMA [10], and the process for the selection of primary studies is described in Figure 2. The first 253 possible research studies were filtered to a final number of 62, and then each study was analyzed to extract information and answer the research questions. The search was carried out on 10 March 2022, on 4 main electronic databases: Scopus, IEEE, ACM, and Web of Science. First, in the identification stage, we used Key1 to obtain 253 primary studies. Because some studies are in more than one electronic database, we implemented a filter to eliminate duplicate studies. This led to 136 primary studies in this stage. In the second stage, called screening, we started with the 136 primary studies obtained in the previous stage and proceeded to filter based on the title of the study, document type, and documents that could not be downloaded, thus reaching 118 possible primary studies. The next step was to analyze these studies through the abstracts and the conclusions to have a better idea of which ones to exclude, so we reached 71 possible primary studies. Finally, the primary studies were analyzed in depth, excluding only 9 that did not align with our field of study (the method of obtaining images was not through cameras; instead, signals and sensors were used). In this way, we had 62 defined primary studies, which are the continuation of this systematic review.

### 2.4. Study Quality Assessment

A quality assessment was performed to select only the most relevant studies for this review in the field of surface defect detection with CNNs. Thus, we developed a questionnaire of nine questions based on the guidelines in [11,12], to analyze the relevance and strength of the primary studies, which were carried out based on the suggestions of the most experienced members of the team. Table 1 shows the quality assessment questions with scores of 1 (yes), 0.5 (partially), and 0 (no). Two researchers analyzed each DDA and answered the quality assessment questions; therefore, the final score is obtained from the average of the sum of the values assigned to each question, considering that the maximum score of a DDA is 9 and the minimum score is 0. The final scores for each question are ranked in the following categories: very high (9≥score>8), high (8≥score>6.5), medium (6.5≥score>4.5), low (4.5≥score>2.5), and very low (2.5≥score≥0).

The list of these 62 selected papers up to this point in the process, the scores of each researcher independently, and the final scores for each primary study can be found in Table A1 located in the Appendix A. After calculating the average scores, we created Table 2 to show the number of studies for each category. The results indicate that most of the studies are in the “Very high” and “High” categories, giving meaning to the filters applied in the previous subsections, emphasizing that 41 studies had the highest scores. To ensure the quality of the obtained results, the team members chose only studies with an average score greater than 4.5 or studies from the “Medium” category onwards, to use in the following sections.

Finally, after several meetings and debates on the exclusion and inclusion of the studies, only DDA12, DDA14, and DDA27 (located in Table A1) were discarded, which obtained the lowest results; therefore, we decided to establish 59 final primary studies (the ones identified in Table 2 from the “Medium” category onwards) as the basis of this systematic review.

## 3. Results

This section shows the results of the literature review with the studies selected in the previous section. The first result is the five-part taxonomy defined for this systematic review, and then Section 3.1 details the results through tables and figures generated throughout this process, and finally, Section 3.2 provides an overview of the applications of CNNs in defect detection.

**Figure 3:**
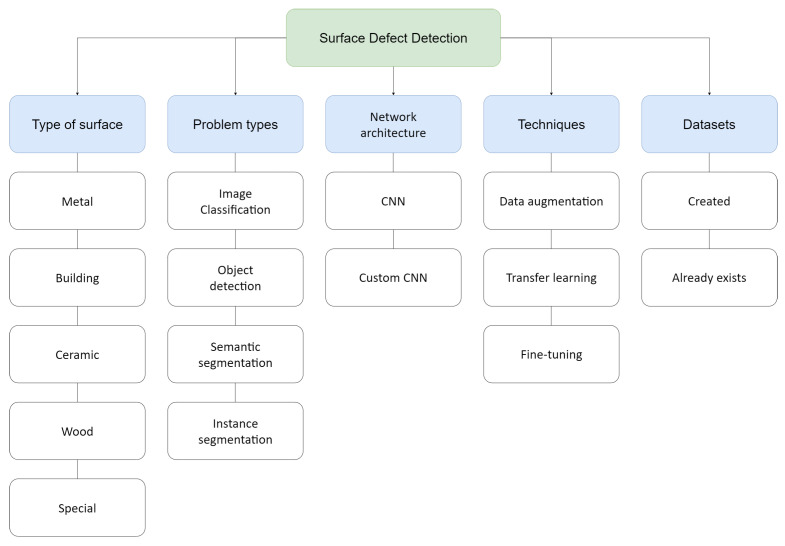
Results taxonomy.

The taxonomy (see Figure 3) is divided into five dimensions: the first dimension (type of surface) refers to the classification of surfaces into five main categories, the second dimension (problem types) is organized into four categories according to problem types with which the networks will be used, the third dimension (network architecture) is divided into two categories according to the network modifications, the fourth dimension (techniques) refers to the most used techniques, and finally, the fifth dimension classifies according to the origin of the dataset.

We refer to learning based on artificial neurons called ANNs, which are large sets of neurons where most neurons are interconnected with each other, literally like our human brain, and they consist of several neurons organized in different layers: an input layer, an output layer, and one or more hidden layers [13]. A deep neural network (DNN) represents an ANN architecture with a greater number of layers between the input layer and output layer; these layers are interconnected to each other and work in parallel [14]. A CNN is a type of DNN that has convolutional layers to reduce the number of training parameters (biases and weights) [1].

Taking into account the number of studies and their surface types, we created five groups that have characteristics in common and were grouped into metal, construction, ceramic, wood, and special. Among the five types of surfaces, special surfaces stand out because they have special characteristics, few studies, and uncommon defects.

Machine learning algorithms include several types based on problem types; among the most common, we defined four categories used for surface defect detection. The first type is classification, whose objective is to accurately identify the features present in an image; thus, the unknown data in an image are classified into predefined classes using a label during training [15]. Then, object detection is based on identifying the location in images or digital videos and then determining to which previously determined class it belongs [16,17]. After that is semantic segmentation, which consists of assigning a previously defined category to each pixel of an image, first dividing an image into several parts or regions called “segments” and then classifying the segments into different classes [18,19]. Finally, instance segmentation is based on the combination of object detection and semantic segmentation; therefore, it allows for the detection of multiple objects as distinct individual instances of the same class, assigning different labels to each one, unlike semantic segmentation which detects multiple objects into a single class [20]. The result of a prediction can be true positive (TP), true negative (TN), false positive (FP), or false negative (FN), so we need metrics to evaluate the performance of a model. The most used in defect detection are accuracy, precision, recall (specificity), f1-score, miss rate, average precision (AP), and mean average precision (mAP). These metrics are used depending on the problem type of learning.

Regarding network architectures, we divide them into two types: unchanged networks, such as AlexNet or ResNet, and networks with modifications in their hidden layers or that have been created specifically for an experiment, called custom networks. The output layer is always modified; therefore, these changes are not considered customization.

As for additional techniques, we detail three of the most used techniques for surface defect detection, which helps to improve the results of training in CNNs. Transfer learning uses previously acquired knowledge when solving problems and uses it in a new problem with similar characteristics [21]. Fine-tuning is a common technique used in transfer learning that uses a pretrained model for a specific task and adjusts or modifies it for a specific new task, so it is similar to transfer learning, with the difference that this technique can retrain all or the last layers using new data [22,23]. Data augmentation is a helpful technique when we have small datasets available because it creates synthetic instances and adds them to the training set, through data warping or oversampling [24].

In industries, datasets are generally proprietary and are not exposed to researchers, due to the costs of generating them, for example, BS5-DET [6], but it is still possible to find datasets already created and for open use, like the DAGM dataset [25] or the COCO dataset [26]. Most free datasets are found in Kaggle [27]. For surface defects, one of the most popular and used is the NEU surface defect database [28], made of six kinds of typical surface defects of the hot-rolled steel strip.

### 3.1. Study Characteristics

This subsection provides a summary of all the information found throughout this systematic review. Each study used is referenced in Table 3, so it is the basis for the used analysis to answer the research questions.

**Figure 4:**
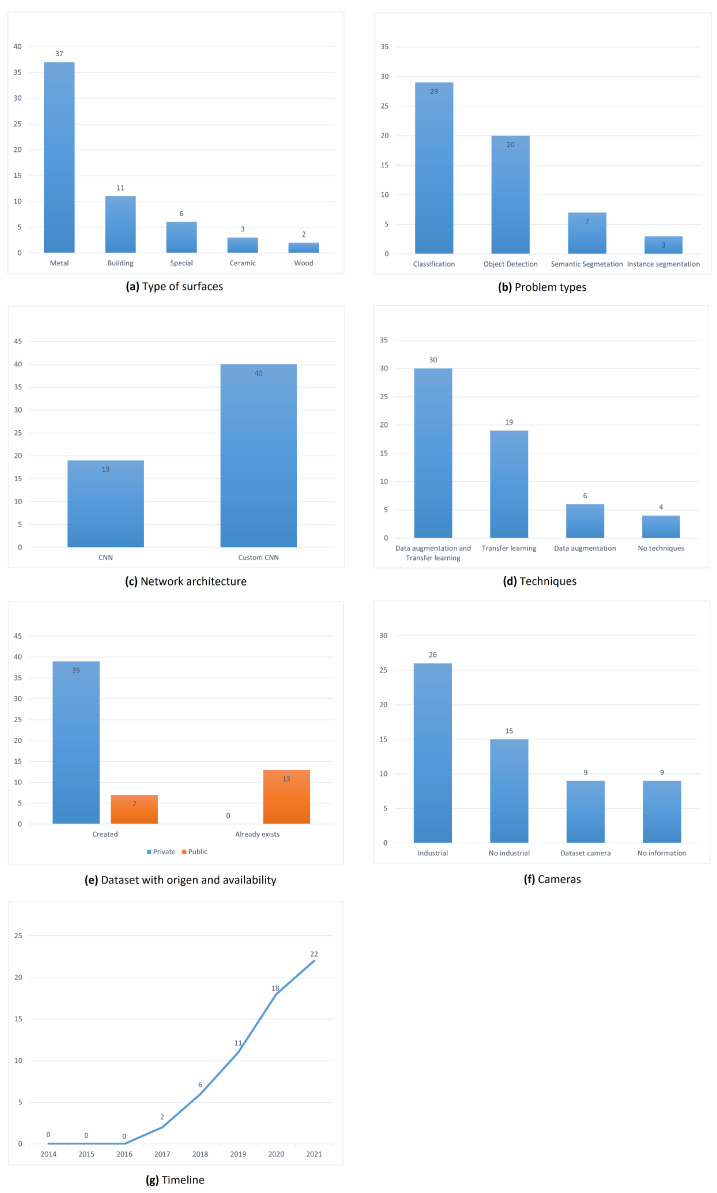
Statistics of study characteristics.

We analyzed and organized the information collected in the studies from Table 3 to find statistics that support our answers to the research questions, so Figure 4 shows seven charts, each chart representing a dimension of the taxonomy and individually showing the details found quantitatively.

The type of surface refers to the number of studies found for this dimension of the taxonomy. Figure 4a shows that metal is the most used surface type in defect detection studies with 37 research studies, and it presents a huge difference compared to other surfaces. In the case of special surfaces, each study shows a surface type that is not easily found in the studies carried out. There are very few studies including these special surface types, and it is because they are not topics that are applied in the industry. Therefore, they do not have a larger budget or an urgent need to be solved but show a field of study that can be explored in the future. Table 4 shows the details of special surface types.

As for the problem types, Figure 4b shows the number of studies and the problem type that was used. We find studies in which only one type of problem is used and others in which the authors use a combination of problem types. In the case of networks, we defined two types of network architecture custom CNNs and traditional CCNs. Custom CNNs have greater use than traditional networks without alterations, as seen in Figure 4c, so this shows that most studies are based on experimenting with new changes in traditional networks to improve results, training times, or use of resources. According to the type of technique used, Figure 4d shows the number of research studies, so most studies use techniques to improve their performance, especially the combination of data augmentation and transfer learning. The datasets have two categories according to their origins, first the datasets that were created as soon as the studies were carried out and then the datasets that had already existed before. Figure 4e shows the number of studies for each type of dataset, so the datasets created were the most used in research studies. This is due to factors such as specificity and the small number of free datasets to carry out studies. Figure 4f shows the number of studies according to the type of camera used. Thus, we were able to identify that industrial cameras are the most used because they have more robust characteristics compared to digital cameras. The timeline presented in Figure 4g shows an increase in the number of studies through the years (the years 2011 to 2021, those considered in the inclusion criteria of this review), which follows the fact that the industry needs to improve quality, and that is why more studies and investment in research began to emerge to help mitigate losses. In 2021, more research studies were carried out than ever.

### 3.2. Applications of CNNs in Defects Detection

This section presents the surface defect detection applications found in this systematic review, grouped according to the taxonomy proposed in this review, specifically for the dimension “type of surface”. To arrive at this relationship, we started by studying the defects of the surfaces and then the most used surfaces in the detection of defects. Thus, five main types of surfaces emerged. The objective is to show the main characteristics of each study as a summary to help researchers who need information on how to detect defects in a type of surface. Metal surfaces are one of the most difficult types for defect detection processes due to the metallic sheen, which affects the visualization of defects. This feature causes visual limitations when performing human eye inspection in industrial manufacturing processes, added to slow detection speed and high labor costs, and makes industries have to look for other alternatives, becoming one of the most studied fields for defect detection [33]. Defect detection in building structures helps us to know the structural stability and prevent structural failure when detected early, so these defects are indicators of aging, decay, or any internal structural fault [73]. Defect detection in ceramic-made products and the porcelain industry is a field of study that has grown in recent years to obtain the benefits of automation, focused on detecting defects such as cracks, bubbles, scratches, and burrs to obtain high-quality control in the industry. So, this type of surface requires delivering top-quality products, because customers are demanding and competition is high; therefore, manual inspection must be improved, at least to reduce material waste [32,82]. Wood is one of the most used engineering materials in the industry and also one of the oldest. Although there are few studies dedicated to this type of surface, its use is still valid and it is exposed to errors in its production, so this type of surface has a large field of study [86]. Finally, according to our analysis and classification, special surfaces are those that are made of unusual materials or materials that have not been studied in depth. These surfaces have singular defects that only occur on these surfaces. Due to the fact of having a small number of studies, they can become a field of study in the coming years. The most important aspects according to the type of surface are presented below in the corresponding tables for each type. Here, the headers P, D, C, and T correspond to the problem type, origin of the dataset, camera, and techniques. P1: image classification, P2: object detection, P3: semantic segmentation, P4: instance segmentation, D1: created, D2: already, C1: industrial, C2: no industrial, C3: dataset camera, C4: no information, T1: transfer learning, T2: data augmentation, T3: no techniques.

#### 3.2.1. Metal

**Figure 5:**
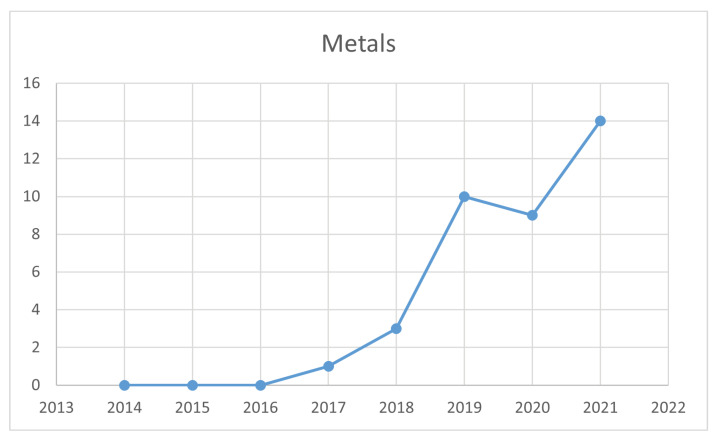
Time line for metals.

Metal is the group with the largest number of studies and is the most used material in industries because metal products are found in our daily lives and industrial production due to their mechanical and physical properties; therefore, failures in metal products not only affect visual characteristics but also characteristics that interfere with the proper functioning of a product. Consequently, these failures cause economic losses in the industry [9]. The rise in research within the metal industry is depicted in Figure 5, underscoring that, despite a decline in 2020 due to the hiatus in activities across most industries during that year, there was a renewed growth in 2021, surpassing all previous years.

The details of Table 5 show that due to a large number of studies and accumulated knowledge about the networks in this type of surface, the trend is to customize the existing networks, because the networks in a simple way already have studies that demonstrate their effectiveness, and the goal is to improve the results or find faster and more efficient methods. YOLOv3 is improved by Shu et al. [39] and Y. Xu, Zhang, et al. [45]. Luo et al. [41] present a method called Smoothing Complete Feature Pyramid Networks (SCFPN), based on FPN, complete intersection over union (CIoU), and label smoothing. Sauter et al. [49] improved VGG16 by eliminating the last layer and replacing it with global average pooling with two dense layers. R. Liu et al. [53] developed a feature refinement Faster R-CNN (FR-FRCNN) based on ResNet. Baskaran and Fernando [57] show a custom MobileNet using GlobalAveragePooling2D and rectified linear unit activation, in addition to using the SGD optimizer with categorical cross entropy loss functionality. Gai et al. [81] presented a custom VGG16 created from the characteristics of ResNet and Inception. Q. Jiang et al. [29] presented a method with a combination of ResNet101 and Faster R-CNN to develop the classification of large images of little objects. Cao et al. [30] present an improved U-Net called SE-U-Net, with two important parts: the SE-Res block and the add operation. Lv et al. [46] proposed an end-to-end defect detection network (EDDN) based on the Single Shot MultiBox Detector, VGG16, and a method called hard negative mining. J. Liu et al. [50] proposed a CNN with batch normalization (BN). Ferguson et al. [72] proposed a defect detection system based on the Mask R-CNN architecture with some parts of ResNet101 and Faster R-CNN; in addition, it is made up of four modules: a feature extraction module, a CNN for a region proposal network (RPN), a CNN for the classification of objects in each RoI, and image segmentation. A framework called MVM-VGG-19 was proposed by Natarajan et al. [84] for anomaly classification that utilizes CNNs with transfer learning together with a mechanism called the majority voting mechanism (MVM).

However, a small group of studies use no customized networks but propose techniques to improve results. To save training time with poor datasets, Lin and Wibowo [33] proposed a comprehensive evaluation score combining defect visibility, visibility distribution, and overexposure based on CNN operating principles. Block et al. [35] proposed a framework based on RetinaNet and minimum output sum of squared error (MOSSE) for tracking. This last part is used to avoid ignoring the temporal coherence between frames and not producing redundant detections for the same defect. Phua and Theng [71] proposed a cascading CNN architecture (DLADC) based on ResNet101 and SSD-VGG16, with the particularity that the authors use the size of the defect as an important indicator in the process. Mittel and Kerber [56] show an automated visual inspection system based on transfer learning, data augmentation, oversampling, and supervised learning with GoogLeNet and AlexNet. Shang et al. [76] proposed two-stage defect detection with Inception v3, transfer learning, and a novel loss function.

Metal surfaces are the group with the most subtypes because metals can be used in their pure state, in alloys such as steel (the most used metal in this systematic review), or in interesting surfaces such as titanium-coated metal [31], microscopic images from thin metal film in electronic components [67], semiconductor wafers (from the metal layers) [71], polishing metal shafts [29], car wiper arms [58], microscopic metal parts [62], cuts from laser cutting machines [64], wind turbine blades [66], insulators in the transmission line aims [68], and X-ray images from metals [72]. Most of the datasets are created by the authors and are kept private; however, some are free to use like BS5-DET [6], CSU_STEEL [41], and GC10-DET [46]. For studies that aim to compare the proposed methods with traditional networks or their datasets such as [41,46,50,53,57,84], the most commonly used option for metal is NEU-DET [28], which has been tested and contains six of the most common defects (crazing, inclusion, patches, pitted surface, rolled-in scale, and scratches).

Each study presents its categories of defects according to the experiments and the characteristics of the material used. Although some are similar, these defects are categorized according to the criteria of the researchers, for example, contusions [6], protrusions [9], abrasions [39], wrinkles [45], rubbing [81], and dents [58]. Among the most common metallic defects are scratches, spots, oxidation, oil droplets, cracks, inclusion, bumps, and cuts. This list of defects, together with the categories of the datasets, is a guide for future studies in this type of material.

As for the techniques used, transfer learning was used in most of the experiments, to take advantage of the knowledge generated previously, marking a trend in the use of this technique for this type of surface. For data augmentation, the use is limited to half of the studies, due to several reasons. On the one hand, there are public datasets with large numbers of images; therefore, it is not necessary to apply the technique. On the other hand, the created datasets use this technique because the metal industry is limited by the rules of factories, which causes difficulties in capturing images for the datasets and makes necessary the use of the data augmentation technique to save time and improve the datasets. Finally, factories invest large amounts of money which, in most cases, allows them to purchase high-end equipment and create specific modules for image capture within the production environment. These modules contain fixed lighting to solve the problem of glare and mostly use industrial cameras due to image quality.

#### 3.2.2. Building

Building surfaces cover several locations such as bridges, pavement, roads, houses, or dams. The most common defects are cracks, but there are others such as intact, spall, or efflorescence [70]. In this type of surface, the authors do not have a bias toward using one type of network architecture; on the contrary, the number of studies for each type of network is almost equal. Y. Xu, Li, et al. [37] proposed an automatic defect detection and segmentation technique based on an improved Mask R-CNN, data augmentation, and transfer learning in tunnel surface images. Kim et al. [47] proposed a novel shallow CNN-based architecture for crack defect detection on concrete surfaces called OLeNet. Mouzinho and Fukai [59] proposed a U-Net-based framework for road surface damages and markings detection on paved roads, to avoid off-road defect detection. Kumar, Sharma, et al. [61] showed a semantic segmentation of concrete surface defects based on Mask R-CNN with transfer learning. Kumar, Batchu, et al. [63] presented a multidrone-based real-time damage detection system (DDS) using the edge computing principle and YOLOv3 for surface concrete damage. Saeed [65] proposed a method for concrete surface defect detection in high places like the pillars of bridges, high-rise buildings, and tall concrete structures, with CNNs. The work presented by Ali et al. [73] shows an automatic inspection system based on CNNs and transfer learning, which consists of using pretrained models and customizing the CNNs. Maningo et al. [79] proposed a crack-detecting system capable of analyzing the physical characteristics of cracks and mapping the surfaces of walls, based on a Faster R-CNN. Zheng et al. [42] present a method for the detection of building cracks based on FCN, R-CNN, and RFCN using semantic segmentation, to detect anomalies in concrete structures. Ahmed et al. [52] created a customized CNN and compared the results between their network and the state-of-the-art Inception-ResNet-v2, Inception-v3, and Xception. N. Wang et al. [70] carried out a study on an interesting surface. It was for Masonry Historic Structures, specifically from orthophotos of the Forbidden City Wall in China.

Creating a dataset becomes a challenge in this type of surface because of the difficulty of access to the site. To achieve a great variety of images, the authors take advantage of different time periods (morning, noon, evening), different shooting distances, light and shadow illuminations, etc. Consequently, the authors use unmanned aerial vehicles for buildings with difficult access such as in [47,61,63,65], or they use ground vehicles for roads such as in [37,52,59]. Moreover, it is the surface where industrial cameras do not stand out; on the contrary, other types of cameras are used, such as Canon (SX60 HS) [63], the Transcend DrivePro 230 camera [59], or smartphone cameras [52], demonstrating that on this surface the important thing is to find the way to access the place to take the picture. These created datasets are mostly kept private, except for the brick/masonry dataset [70], created and available online, along with the code to replicate the project. On the other hand, there are online public datasets such as the Middle East Technical University (METU) dataset [47], the Kaggle library [73], and the SDNET2018 dataset [79], which contain thousands of checked images and are used to demonstrate whether the proposed method obtains good results. Ahmed et al. [52] use the Cityscapes and KITTI road datasets to compare with the dataset created by the authors, which is a practice with excellent results.

As shown in Table 6, in most of the studies, it was not necessary to use data augmentation, because the building industry is not limited to a factory, so image capture depended mostly on the researchers of the proposed methods. The methods created by the authors facilitated the capture of images, and in other cases, public datasets containing thousands of images were used; however, the use of transfer learning remains a constant in most of the studies, taking advantage of the knowledge acquired by previously trained networks.

#### 3.2.3. Ceramic

Defect detection in the ceramics industry is aimed at reducing manufacturing time and increasing production efficiency by avoiding the release of defective products to the market. As it is an industry with few studies, most of them use networks already created and compare them. Min et al. [32] proposed the use of CNNs (ResNet20, ResNet56, and ResNet110) in defect detection for ceramic images with data augmentation. Karangwa et al. [40] present a proposal for surface defect detection based on a Faster R-CNN with VGG16. However, with custom CNNs, Birlutiu et al. [82] presented an automated defect management system with real-time high-speed processing to classify and predict images with and without defects.

Defects like breaks, cracks, pinholes, dirt, pits, and spots, shown in [40], are repeated in this material; therefore, they can be used as a guide for future studies. The datasets have not been released because we work with factories, so the information is kept private. Likewise, we did not obtain much information on the cameras used and lighting, but [40] detailed how the authors solved the problem of lighting in highly reflective materials with a light source intensity controller and coaxial lights to create a lighting system. In terms of techniques, there is a tendency to use a combination of data augmentation and transfer learning, as shown in Table 7.

#### 3.2.4. Wood

The wood industry is a good place to conduct studies because wood has the characteristic of creating randomly textured surfaces, which is an advantage when using data augmentation techniques. In a study conducted by Jung et al. [78], the authors propose a technique to create randomly textured surfaces, augmenting their dataset with up to 10,000 images divided into five classes (dye, adhesives, oil, scratch, and normal or defect-free), overcoming the problems of missing images, dataset imbalance, overfitting, and underfitting. To test their technique, the authors used LeNet, VGG19, and Densenet121 with transfer learning, achieving accuracy values of 95.00%, 99.80%, and 98.90%, respectively, without many drawbacks.

As in most studies, in [44], a custom network for defect detection with an improved SSD is proposed. This proposal modifies the SSD algorithm, internally replacing the VGG16 network with a DenseNet121, together with the transfer learning technique. For the dataset, the authors use image acquisition equipment, with industrial cameras and controlled lights on a walkway, collecting 400 images, which were cut, segmented, and resized, arriving at an initial dataset of 500 images of wood knots, dead knots, and checking defects. Then, the dataset was improved to over 2000 images with data augmentation techniques. This study achieved a mean average precision of 96.1%, which was superior to the compared methods during the experiments.

These two research papers demonstrate that a surface with few studies should use transfer learning and data augmentation techniques to improve results. Furthermore, in both cases, the authors were found to create their datasets with industrial cameras to improve image quality. These details are shown in Table 8.

#### 3.2.5. Special

These surfaces stand out for their unique qualities, and most use customized nets to achieve their objectives. Zou et al. [43] proposed an improved U-Net for defect detection on colored paintings on the surfaces of ancient Chinese buildings, to help restorers with a reference and orientation of how the paintings looked before the weathering process, because this is repetitive work that takes considerable time. J. Jiang et al. [83] proposed a novel inspection system for manufacturing mobile phone back glass (MPBG), based on a modified segmentation DCNN. Tabernik et al. [36] proposed a segmentation-based DCNN based on a two-stage architecture for detecting surface-crack defects in industrial processes specifically on the surface of the plastic embedding in electrical commutators. Y. Li et al. [80] proposed a method called MobileNet-SSD to identify the types and locations of defects such as breaches, dents, burrs, and abrasions on the sealing surface of a container in the filling line. Furthermore, some studies do not alter the existing nets and use several to check the results. F. Xu et al. [51] proposed a method for defect detection in paint film for anticorrosion and decoration of metal workpieces, based on SSD and Faster R-CNN with data argumentation techniques. Le et al. [34] presented a proposal for the detection of defects in small databases based on data augmentation, transfer learning, and multimodel ensemble for decorative sheet and welding defects, with the distinctive feature that the latter defects are X-ray images.

As detailed in Table 9, regarding techniques, these studies use transfer learning, data augmentation, or a combination of both. However, only [83] did not use the data augmentation technique, due to a coaxial bright-field (CBF) imaging system and a low-angle bright-field (LABF) imaging system proposed by the authors which captured more than 10,000 images. In this type of surface, industrial cameras continue to be the most widely used; however, Zou et al. [43] captured the images with a smartphone camera, achieving a good image quality. The datasets created are generally private; only KolektorSDD [36] is for public use.

## 4. Discussion

This section presents the results gathered from the analysis of the primary studies for surface defect detection. We present the answers to the research questions in each subsection, including an analysis and discussion of each one, considering the information obtained throughout the systematic review and the results of Section 3. We also highlight the main lessons learned in this review.

### 4.1. Research Questions

We made a more detailed review of the results gathered in Section 3 to answer the research questions. In this way, we obtained answers and trends that have been marked in recent years for surface defect detection.

#### 4.1.1. RQ1: Which Are the Most Used Types of Surfaces in Defect Detection?

In the first instance, there is a significant difference between the surfaces used, as presented in Table 10, which shows the total number of studies for each type of surface. Metal is the type of surface with the most studies carried out, at 62.71%. This is because metal and its derivatives are widely used materials in world industry, despite the difficulty involved in studying this material because lighting and its reflection have been a challenge in most of the studies. The second type of surface with the highest number of studies is building surfaces, at 18.64%. Although there are few studies, these are important because most of them are used as a method to prevent damage or catastrophes, but even so, the difference between it and the metal industry is wide. Special surfaces as a whole achieved 10.17%, demonstrating that research into new types of materials is growing. Ceramic and wood surfaces are common types of surfaces but do not have a considerable number of studies yet, which indicates that they are good fields for future research.

#### 4.1.2. RQ2: What Are the Main Problem Types for Surface Defect Detection?

According to problem types, we categorized four types:

- P1: image classification;
- P2: object detection;
- P3: semantic segmentation;
- Instance segmentation.

To find the most used problem types, Table 11 shows the percentages of use of each type. Here, we found that image classification is the most used problem type, with 49.15% of studies using this type. This difference in percentages between classification and the other methods is because most of the deep learning methods are made for this type of data; in addition, the computational cost is the lowest and there is enough information to carry out experiments or consult. Then, object detection, in 33.90% of the studies, is used to detect the place where the defects are located, which gives more information to researchers than using it but requires more computational resources. Finally, semantic segmentation at 11.86% and instance segmentation at 5.08% are the types with the least use. This reduction in the percentages is because their computational cost is quite high compared to the other types; also, the cost of economic resources is higher, which makes researchers and industries think twice before deciding on these algorithms, although their level of detail is quite high and gives more information to make decisions.

In this systematic review, we account for the problem types that have the greatest impact on each study. However, there are also studies where more than one type is used, as in the case of [71,76], where the authors combine classification and object detection, or in the case of study [36], which uses a combination of semantic segmentation and classification. These combinations occur because the authors make comparisons with several models or create modules or phases in their proposals, therefore requiring more than one problem type to better show their results.

For image classification challenges, exploring alternatives like graphs is valuable. In [87], a fresh approach using multiple graph learning neural networks (MGLNN) for classification is presented. This method employs semisupervised learning and multiple datasets, including Caltech101-7 [88] with 1474 images. The current challenge revolves around the issue of bounding box noise in object detection networks. Consequently, one of the most extensively studied approaches is exemplified in [89], where the authors propose a solution termed DenseSPH-YOLOv5. This solution incorporates convolutional block attention modules (CBAMs) to enhance real-time performance. These focused point-wise amalgamations delineate an emerging frontier of exploration within CNNs.

The ongoing challenge in semantic and instance segmentation is the computational cost, which motivates researchers to concentrate on finding solutions. A starting point could be the analysis of performance under hardware limitations presented in [90] or the survey presented in [91], where the issue of computational cost is tackled. Subsequently, it is valid to explore proposals such as [92,93,94], in which authors introduce innovative modules, network adjustments, and methodologies aimed at alleviating the computational burden.

#### 4.1.3. RQ3: Which Is the Type of Network Architecture Most Used for Each Type of Surface Defect Detection?

Given the plethora of variations within network architectures, our focus lies in categorizing them into two main types: CNNs and custom CNNs. A CNN entails a traditional convolutional neural network devoid of alterations. Typically, comparative studies involving datasets or techniques devised by researchers are employed to enhance outcomes. Conversely, a custom CNN pertains to a personalized convolutional neural network that undergoes structural alterations or modifications. In certain instances, authors enhance these networks by crafting novel modules or amalgamating components from various network architectures to engender a novel network configuration.

The difference between the studies that created their own CNNs and those that used state-of-the-art networks to carry out the experiments is shown in Table 12. Therefore, 67.80% of the studies created a new CNN based on other CNNs that already exist or they also customized a CNN with a few modifications to make it faster or more accurate. On the contrary, only a third of the studies used traditional or existing networks, which shows the tendency to make modifications to find better results.

#### 4.1.4. RQ4: What Techniques Were Used to Improve Performance in Studies Regarding Surface Defect Detection?

According to the types of techniques, we categorized two types:

- DA: data augmentation;
- TL: transfer learning.

The number of studies using transfer learning, data augmentation, or a combination of both to improve the performance of the models is summarized in Table 13. An interesting fact is that only 6.78% of the studies do not use these techniques or the authors do not give details about the technique used, which shows us that most researchers use at least one of these two techniques. The combination of transfer learning and data augmentation has the highest percentage of utilization, at 50.85%, because most studies use this combination to improve results. Then, 32.20% of studies use only transfer learning and 10.17% of studies use only data augmentation. A few studies use only one of these two techniques, unlike the combinations that are widely used for the defect detection process. Therefore, we conclude that to obtain the best results, most authors use the combination of transfer learning and data augmentation.

To find out which is the most used technique, we are guided by Table 14, which shows the number of studies where each technique was used, regardless of whether it was used in a combination or individually. Transfer learning is the most used technique, with a percentage of 83.05%, and data augmentation follows, being used in 59.32% of the studies. These percentages are high due to the benefits of using pretrained models or performing data augmentation techniques when our datasets are small. Transfer learning has the highest percentage because a few studies train their neural networks without the use of a pretrained model, but for data augmentation, the percentage decreases because the authors have the possibility of capturing images in the modules that they create and implement or because public datasets can have a large number of images, which do not require an increase in data.

#### 4.1.5. RQ5: What Type of Dataset Is the Most Used?

The origin and availability of datasets are important parameters when starting experiments with defect detection. Table 15 shows the origin of datasets, so 77.97% of the datasets were created from cameras and 22.03% used datasets previously created, which are generally downloaded or obtained from other similar studies. This means that most experiments need to create their datasets because there are few options available in digital repositories, and these available datasets are not similar to the type of surface needed by researchers. Regarding availability in Table 16, the datasets created above are generally available in digital repositories and are in public use, which is a great help for researchers. The most used public datasets are the Kaggle library [73], KITTI [95], SDNET2018 [79], GDXray [72], and NEU [28] which is the dataset that was referred to more often in this systematic review.

According to Table 17, only a few of the datasets created are public. This is because industries invest money and time and they prefer to reserve their information privately. In numerical terms, from the 45 datasets created, only 7 studies are available in public repositories. These datasets are ALCIDE [64], BS5-DET [6], CSU_STEEL [41], GC10-DET [46], KolektorSDD [36], and the datasets created in [35,70].

In this systematic review, the studied datasets primarily employ cameras for image capture. There are instances where existing datasets alone are used and others where a combination of precreated datasets and camera images are employed for defect detection experiments. Table 18 provides an overview of the study count and the types of cameras utilized. Industrial cameras take the lead at 44.07%, primarily due to their superior resolutions compared to other camera types. It is important to note that despite lacking autofocus capabilities in many cases, industrial cameras heavily rely on specific lighting conditions and specialized lenses, explaining their prevalence. Subsequently, nonindustrial cameras account for 25.42%. Within this category, prevalent nonindustrial cameras encompass smartphone cameras, USB cameras, drone cameras, and even common-use cameras. Merely 15.25% of studies exclusively conduct experiments using dataset images, primarily focusing on neural network comparisons. Conversely, 15.25% of studies acknowledge camera usage, albeit without furnishing detailed specifications regarding camera types or attributes. This limitation hinders the direct applicability of these studies within industry contexts. Notably, some authors choose to retain proprietary information, and certain companies restrict the dissemination of images, consequently constraining their suitability for real-world applications.

#### 4.1.6. RQ6: How Did the Number of Studies Evolve over the Years?

The search key begins with studies from the year 2011, and the first two studies appear in the year 2017, while in the previous years, no studies were found, according to our filtering in Section 2. Since then, the number of studies has begun to increase. This increase is due to the continuous improvement of algorithms and techniques for the detection of defects. Thus, Table 19 shows that the year with the most studies was 2021, at 37.29%.

### 4.2. Learned Lessons

The review at hand encompassed a meticulous examination of numerous works within the chosen literature. Over the course of this systematic review, a predominant concern that emerged was the insufficiency of information in specific studies. Among the most notable aspects, information pertaining to lighting, quantity of images, and image dimensions stood out. Gathering this information proved to be a challenge; nevertheless, it served as a valuable learning tool that could drive the execution of further systematic reviews of this nature in the future. The obtained results and the collected information allowed us to arrive at a set of insights that translate into several lessons learned. Therefore, the main learned lessons are the following:

- In industry, metal surfaces are the most used, being in 62.71% of primary studies, even though this type of surface is difficult to study because the light is reflected and it is not easy to obtain superior-quality datasets at the beginning;
- According to problem types, image classification is the most used type of learning individually or in combination, because there is a lot of information and its computational cost is less high than the other problem types. It is followed by object detection and finally by semantic segmentation together with instance segmentation, which have the highest computational cost and take the longest time to compute;
- Using techniques to improve performance is common in this type of study, due to the difficulty of creating datasets with large numbers of images. A total of 93.22% of the studies use at least one technique to improve performance; it can be transfer learning or data augmentation. Individually, transfer learning is the most popular among researchers;
- The number of studies conducted on surface defect detection with CNNs is increasing every year because it provides better results in the industry, helps reduce costs, and increases the speed of production when implemented in a factory. These technological solutions not only offer these benefits but also have the potential to bring about significant changes in the industrial sector. By harnessing these advancements, businesses can gain a substantial competitive edge over their counterparts;
- To create datasets, industrial cameras are the most used and showed better results due to their ability to capture better-quality images than conventional cameras or web cameras. However, in conditions of difficult access to study sites, the authors used various types of cameras;
- The traditional networks have already been tested with several experiments and studies. However, to obtain more and more accurate results, current studies are focused on modifying these networks or creating complementary methods to improve defect detection. We note that this trend is growing, especially on surfaces with the largest number of studies.

## 5. Conclusions and Future Work

In this paper, we present a systematic literature review for surface defect detection using CNNs with supervised learning. First, we carried out an analysis of the main databases, defining 59 primary studies between 2010 and 2021. Secondly, we summarized the studies and extracted the main characteristics such as type of surface, problem type, timeline, type of network, techniques, and datasets. Finally, we compared and analyzed the information gathered.

The use of CNNs for surface defect detection is increasing every year. Even though the metal industry is the one that invests the most in these studies, other industries are beginning to investigate due to the satisfactory results that have been shown, and so in the future, they will continue to be excellent fields of study for future research and applications in the industry. In addition, computers are becoming more powerful and accessible, allowing researchers to perform more complex processing tasks, taking less time to obtain results. Therefore, although Classification is the most used problem type, in the coming years, projects could be carried out that combine the four types (classification, object detection, semantic segmentation, and instance segmentation) to obtain more precise results that improve the quality of the products.

We observed that in about half of the studies, there is no information provided regarding the cameras, lighting, or image size used. Specifically, we encounter challenges when searching for information that explains how they address lighting challenges in both controlled environments and those with natural light. This becomes critically important because in real industry settings, products are often manufactured in warehouses or spaces with natural lighting to save costs. Therefore, this aspect is vital for improving image quality, especially when cameras lack autofocus. All of this information becomes crucial when using a study as a reference in developing a practical application aimed at defect detection. However, we are faced with the difficulty of finding studies that offer the necessary guidance for creating effective applications in the industry.

The following guidelines were made for future researchers and professionals interested in this field of study:

- Researchers must diligently screen articles containing extensive information on image capture. Often, in this domain, data from one source can be reused in another, making data reuse feasible. In this scenario, it is noteworthy that only 15.25% of the studies did not reveal information about the use of cameras for their datasets. Therefore, existing modules created for image capture can be used as a guide;
- Some studies withhold relevant information within their datasets, especially the quantity of generated images. This omission restricts essential data access for researchers or professionals in need of using such information for real-world applications or comparing new network architectures. Therefore, utilizing existing datasets as a guide for constructing our dataset proves to be a prudent approach;
- Researchers about to conduct flaw detection studies must first focus on the type of surface they are going to study. If there is no information regarding the surface sought, similar surfaces must be used because defects are repeated on most surfaces;
- Researchers who possess limited experience in this field should initiate their endeavors by conducting experiments on metal surfaces, leveraging the wealth of existing data. Subsequently, they can transition to their specific area of interest or the surface type they are studying.

## Figures and Tables

**Table 1:** Quality assessment (QA) questions.

#Q	Quality Questions	Yes	Partially	No
QA1	Are the objectives of the study clearly identified?			
QA2	Are the limitations of the study specified?			
QA3	Is the type of surface specified and characterized?			
QA4	Does the study have a description and characterization of the used technology?			
QA5	Is it clear how the data collection was performed for the datasets?			
QA6	Is the dataset size appropriate?			
QA7	Are the findings and results correctly declared and discussed?			
QA8	Is the research methodology repeatable?			
QA9	Was a comparative analysis conducted (algorithm types)?			

**Table 2:** Studies per category.

#	Rank	Category	Studies
1	9≥score>8	Very high	6
2	8≥score>6.5	High	35
3	6.5≥score>4.5	Medium	18
4	4.5≥score>2.5	Low	2
5	2.5≥score≥0	Very low	1

**Table 3:** Summary.

Reference	Author	Reference	Author
[6]	Kou et al.	[29]	Q. Jiang et al.
[9]	He et al.	[30]	Cao et al.
[31]	Aslam et al.	[32]	Min et al.
[33]	Lin and Wibowo	[34]	Le et al.
[35]	Block et al.	[36]	Tabernik et al.
[37]	Y. Xu, Li, et al.	[38]	Lian et al.
[39]	Shu et al.	[40]	Karangwa et al.
[41]	Luo et al.	[42]	Zheng et al.
[43]	Zou et al.	[44]	Ding et al.
[45]	Y. Xu, Zhang, et al.	[46]	Lv et al.
[47]	Kim et al.	[48]	K. Li et al.
[49]	Sauter et al.	[50]	J. Liu et al.
[51]	F. Xu et al.	[52]	Ahmed et al.
[53]	R. Liu et al.	[54]	Ren et al.
[55]	Feng et al.	[56]	Mittel and Kerber
[57]	Baskaran and Fernando	[58]	Ooi et al.
[59]	Mouzinho and Fukai	[60]	J. Sun et al.
[61]	Kumar, Sharma, et al.	[62]	Han et al.
[63]	Kumar, Batchu, et al.	[64]	Santolini et al.
[65]	Saeed	[66]	Zhao et al.
[67]	Kamiyama et al.	[68]	Guo et al.
[69]	Mao et al.	[70]	Wang et al.
[71]	Phua and Theng	[72]	Ferguson et al.
[73]	Ali et al.	[74]	W. Sun et al.
[75]	Zhou et al.	[76]	Shang et al.
[77]	Bahrami et al.	[78]	Jung et al.
[79]	Maningo et al.	[80]	Y. Li et al.
[81]	Gai et al.	[82]	Birlutiu et al.
[83]	J. Jiang et al.	[84]	Natarajan et al.
[85]	Yun et al.		

**Table 4:** Specifics of special surfaces.

Reference	Surface	Details
[43]	Special	Colored paintings on the surfaces of ancient Chinese buildings
[51]	Special	Paint film to protect and decorate metallic workpieces
[83]	Special	Mobile phone back glass defects
[34]	Special	Decorative sheets and welding defects
[36]	Special	Plastic embedding defects in electrical commutators
[80]	Special	Sealing surface defect of a container in the filling line

**Table 5:** Details of metal surfaces.

Reference	Problem	Dataset	Camera	Technique	Year	Network Architecture
[6]	P2	D1	C4	T1-T2	2021	Custom R-CNN
[9]	P1	D1	C1	T2	2021	ResNet, DenseNet
[31]	P3	D1	C4	T1	2021	Custom U-Net
[33]	P2	D1	C1	T1-T2	2021	YOLO, SDD, Faster R-CNN
[35]	P2	D1	C1	T1	2021	RetinaNet
[39]	P2	D1	C4	T1-T2	2021	Custom YOLOv3
[41]	P2	D1	C1	T1	2021	Custom FPN
[45]	P2	D2	C1	T1-T2	2021	Custom YOLOv3
[49]	P1	D2	C1	T1-T2	2021	Custom VGG16
[53]	P2	D2	C3	T1	2021	Custom Faster R-CNN
[55]	P1	D1	C4	T2	2021	Custom Xception
[57]	P1	D2	C3	T1-T2	2021	Custom MobileNet
[67]	P1	D1	C2	T1-T2	2021	Custom VGG19
[69]	P1	D2	C3	T1	2021	Custom ResNet
[71]	P1	D1	C2	T1-T2	2020	ResNet, SSD-VGG16
[75]	P1	D1	C1	T1	2020	Compact CNN
[77]	P2	D1	C2	T1	2020	Faster R-CNN, SSD, Inception v2
[81]	P1	D1	C1	T1-T2	2020	Custom VGG
[85]	P1	D1	C1	T2	2020	Custom CNN
[29]	P2	D1	C1	T1	2020	Custom CNN
[30]	P3	D2	C3	T1-T2	2020	Custom SE-U-Net
[38]	P1	D1	C1	T2	2020	Custom CNN
[46]	P2	D1	C1	T1	2020	EDDN
[48]	P2	D2	C3	T1-T2	2019	Custom Faster R-CNN and FPN
[50]	P1	D2	C3	T3	2019	Custom with BN
[54]	P2	D1	C1	T1-T2	2019	Custom Slighter Faster R-CNN
[56]	P1	D1	C2	T1-T2	2019	GoogLeNet, AlexNet
[58]	P1	D1	C4	T2	2019	Custom CNN
[60]	P1	D1	C1	T1-T2	2019	Custom VGG16
[62]	P1	D1	C2	T1-T2	2019	Custom Inception v4
[64]	P1	D2	C3	T1	2019	Custom CNN
[66]	P1	D1	C1	T2	2019	AlexNet, BP neural network
[68]	P2	D1	C4	T1	2019	YOLOv3
[72]	P4	D2	C1	T1-T2	2018	Custom CNN
[74]	P1	D1	C1	T1-T2	2018	Custom CNN
[76]	P2	D1	C1	T1	2018	Inception v3
[84]	P1	D2	C2	T1	2017	Custom VGG

**Table 6:** Details of building surfaces.

Reference	Problem	Dataset	Camera	Technique	Year	Network Architecture
[37]	P4	D1	C1	T1-T2	2021	Custom Mask R-CNN
[47]	P1	D2	C3	T1	2021	Custom LeNet-5
[59]	P3	D1	C2	T1-T2	2021	U-Net
[61]	P4	D1	C2	T1	2021	Mask R-CNN
[63]	P2	D1	C2	T1	2021	YOLOv3
[65]	P1	D1	C2	T3	2021	Custom CNN
[73]	P1	D2	C2	T1-T2	2020	Custom CNN
[79]	P2	D2	C3	T1	2020	Faster R-CNN
[42]	P3	D1	C2	T1	2020	FCN, R-CNN, and RFCN
[52]	P1	D1	C2	T3	2019	Custom CNN, Inception-ResNet-v2, Inception-v3, and Xception
[70]	P2	D1	C2	T1-T2	2018	AlexNet for MHSD, GoogLeNet for MHSD

**Table 7:** Details of ceramic surfaces.

Reference	Problem	Dataset	Camera	Technique	Year	Network Architecture
[32]	P1	D1	C4	T1-T2	2020	ResNet
[40]	P2	D1	C1	T1-T2	2020	Faster R-CNN with VGG16
[82]	P1	D1	C4	T3	2017	Custom CNN

**Table 8:** Details of wood surfaces.

Reference	Problem	Dataset	Camera	Technique	Year	Network Architecture
[44]	P2	D1	C1	T1-T2	2020	Custom SSD
[78]	P1	D1	C1	T1-T2	2018	LeNet, VGG19, DenseNet121

**Table 9:** Details of special surfaces.

Reference	Problem	Dataset	Camera	Technique	Year	Network Architecture
[43]	P3	D1	C2	T1-T2	2021	Custom U-Net
[51]	P2	D1	C1	T1-T2	2021	SSD and Faster R-CNN
[83]	P3	D1	C1	T1	2020	Custom U-Net
[34]	P1	D1	C1	T1-T2	2020	MobileNet, Inception
[36]	P3	D1	C4	T1-T2	2019	Custom CNN
[80]	P2	D1	C1	T1-T2	2018	Custom MobileNet-SSD

**Table 10:** Total of types of surfaces.

Surface	Total	Percentage
Metal	37	62.71%
Building	11	18.64%
Special	6	10.17%
Ceramic	3	5.08%
Wood	2	3.39%

**Table 11:** Individual percentages for use in studies for each type.

Problem Type	Total	Percentage	Details
P1	29	49.15%	Studies using image classification
P2	20	33.90%	Studies using object detection
P3	7	11.86%	Studies using semantic segmentation
P4	3	5.08%	Studies using instance segmentation

**Table 12:** Custom and noncustom networks.

Network	Total	Percentage	Details
CNN	19	32.20%	Studies that used unmodified networks to perform the experiments
Custom CNN	40	67.80%	Studies that created a CNN based on other networks

**Table 13:** Studies with data augmentation and transfer learning.

Technique	Total	Percentage	Details
DA	6	10.17%	Studies that use only data augmentation
TL	19	32.20%	Studies that use only transfer learning
DA and TL	30	50.85%	Studies that use a combination of data augmentation and transfer learning
No technique	4	6.78%	Studies that do not use these techniques

**Table 14:** Use of techniques.

Technique	Total	Percentage	Details
TL	49	83.05%	Studies that use data augmentation
DA	36	59.32%	Studies that use transfer learning

**Table 15:** Origin of datasets.

Origin	Total	Percentage
Created	46	77.97%
Already exists	13	22.03%

**Table 16:** Availability of all datasets.

Origin	Total	Percentage
Private	39	66.10%
Public	20	33.90%

**Table 17:** Availability of created datasets.

Availability	Total	Percentage
Private	39	86.67%
Public	7	13.33%

**Table 18:** Camera types for each study.

Camera	Studies	Percentage
Industrial	26	44.07%
Nonindustrial	15	25.42%
Camera dataset	9	15.25%
No information	9	15.25%

**Table 19:** Total of types of surfaces.

Year	Total	Percentage
2017	2	3.39%
2018	6	10.17%
2019	11	18.64%
2020	18	30.51%
2021	22	37.29%

## Data Availability

In accordance with our research collaboration and data confidentiality agreement, the data used in this study are considered private and cannot be publicly shared. As such, we are unable to provide access to the datasets analyzed or generated during the research. We assure that the privacy and confidentiality of the data were strictly maintained throughout the study, adhering to ethical and legal considerations. While we are unable to make the data publicly available, we have followed the necessary protocols to ensure the integrity and validity of our findings.

## Appendix A

In this appendix section, the answers to the quality questions stated in Table 1 are presented (in Table A1).

**Table A1:** Final quality scores of answers for each DDA.

Study No.	Reference	Score 1	Score 2	Final Score	Study No.	Reference	Score 1	Score 2	Final Score
DDA1	[6]	7	8	7.5	DDA32	[83]	7.5	8.5	8
DDA2	[9]	4.5	6	5.25	DDA33	[85]	8.5	6.5	7.5
DDA3	[31]	7.5	6	6.75	DDA34	[29]	9	7.5	8.25
DDA4	[33]	8	9	8.5	DDA35	[30]	7.5	7.5	7.5
DDA5	[35]	8.5	8.5	8.5	DDA36	[32]	7	6.5	6.75
DDA6	[37]	8	8	8	DDA37	[34]	8	7.5	7.75
DDA7	[39]	8.5	8.5	8.5	DDA38	[36]	8	8	8
DDA8	[41]	7	7	7	DDA39	[38]	7	8	7.5
DDA9	[43]	6	5.5	5.75	DDA40	[40]	6.5	6	6.25
DDA10	[45]	5.5	5.5	5.5	DDA41	[42]	8.5	8.5	8.5
DDA11	[47]	7.5	8	7.75	DDA42	[44]	8	8	8
DDA12	[86]	4	3.5	3.75	DDA43	[46]	8	8	8
DDA13	[49]	6	6	6	DDA44	[48]	7	8	7.5
DDA14	[96]	2	3	2.5	DDA45	[50]	5	5.5	5.25
DDA15	[51]	7	7	7	DDA46	[52]	6	5	5.5
DDA16	[53]	7	7	7	DDA47	[54]	8.5	8.5	8.5
DDA17	[55]	6.5	6.5	6.5	DDA48	[56]	7	7.5	7.25
DDA18	[57]	5.5	5.5	5.5	DDA49	[58]	6.5	6.5	6.5
DDA19	[59]	7	6.5	6.75	DDA50	[60]	6	7.5	6.75
DDA20	[61]	8	8	8	DDA51	[62]	6.5	6.5	6.5
DDA21	[63]	6.5	6	6.25	DDA52	[64]	7.5	7.5	7.5
DDA22	[65]	6.5	6.5	6.5	DDA53	[66]	4	8	6
DDA23	[67]	6.5	7	6.75	DDA54	[68]	3.5	6	4.75
DDA24	[69]	6.5	7	6.75	DDA55	[70]	7.5	7.5	7.5
DDA25	[71]	5.5	6.5	6	DDA56	[72]	7.5	7.5	7.5
DDA26	[73]	7	8.5	7.75	DDA57	[74]	8	8	8
DDA27	[97]	2.5	5	3.75	DDA58	[76]	8	8	8
DDA28	[75]	7.5	8.5	8	DDA59	[78]	8	7.5	7.75
DDA29	[77]	5.5	8.5	7	DDA60	[80]	8	8	8
DDA30	[79]	5.5	6.5	6	DDA61	[82]	7.5	7.5	7.5
DDA31	[81]	5	5.5	5.25	DDA62	[84]	7.5	7.5	7.5
